# Alcohol consumption in relation to cardiovascular and non-cardiovascular mortality in an elderly male Chinese population

**DOI:** 10.1186/s12889-021-12140-6

**Published:** 2021-11-09

**Authors:** Xiao-Fei Ye, Chao-Ying Miao, Wei Zhang, Chang-Sheng Sheng, Qi-Fang Huang, Ji-Guang Wang

**Affiliations:** 1 School of Public Health, Shanghai Jiao Tong University School of Medicine, Shanghai, China; 2 Department of Cardiovascular Medicine, The Shanghai Institute of Hypertension, Ruijin Hospital, Shanghai Jiao Tong University School of Medicine, Shanghai, China

**Keywords:** Alcohol consumption, Mortality, All-cause mortality, Cardiovascular mortality

## Abstract

**Background:**

We investigated the association of alcohol consumption with cardiovascular and non-cardiovascular mortality in elderly Chinese men.

**Methods:**

Our participants were recruited from residents living in a suburban town of Shanghai (≥60 years of age, *n* = 1702). Alcohol intake was classified as non-drinkers, past drinkers (stopped drinking for ≥12 months), and current light-to-moderate (1 to 299 g/week) and heavy drinkers (≥300 g/week). Alcoholic beverages were classified as beer/wine, rice aperitif and liquor/mix drinking.

**Results:**

During 5.9 years (median) of follow-up, all-cause, cardiovascular and non-cardiovascular deaths occurred in 211, 98 and 113 participants, respectively. The corresponding incidence rates were 23.6/1000, 10.9/1000 and 12.6/1000 person-years, respectively. Both before and after adjustment for confounding factors, compared with non-drinkers (*n* = 843), past drinkers (*n* = 241), but not the current light-to-moderate (n = 241) or heavy drinkers (*n* = 377), had a higher risk of all-cause (adjusted hazard ratio [HR] 1.90, 95% confidence interval [CI] 1.35–2.68, *P* = 0.0003) and non-cardiovascular mortality (HR 2.46, 95% CI 1.55–3.91, *P* = 0.0001). Similar trends were observed for cardiovascular mortality (HR 1.44, 95% CI 0.85–2.44, *P* = 0.18). In similar unadjusted and adjusted analyses, compared with the current beer/wine drinkers (*n* = 203), liquor/mix drinkers (*n* = 142), but not aperitif drinkers (*n* = 273), had a significantly higher risk of all-cause (HR 3.07, 95% CI 1.39–6.79, *P* = 0.006), and cardiovascular mortality (HR 10.49, 95% CI 2.00–55.22, *P* = 0.006). Similar trends were observed for non-cardiovascular mortality (HR 1.94, 95% CI 0.73–5.16, *P* = 0.18).

**Conclusions:**

Our study showed risks of mortality associated with past drinking and liquor drinking in the elderly Chinese men.

## Introduction

There is consensus that alcohol intake at any quantity and of any class is detrimental [1]. Indeed, according to the Global Burden of Disease (GDB) study, alcohol intake was associated with 5.3, 3.3 and 4.1% of increased all-cause and cardiovascular mortality and cancers worldwide, respectively. The corresponding increases in all-cause mortality and cancers in the Chinese population were 4.1 and 6.2%, respectively [2, 3]. However, for now and probably for a long while in the future, alcohol drinking will continue as a major lifestyle factor in many populations. In China, alcohol intake is a habit mainly in men and rarely in women, and alcoholic beverages include beer and wine as in other populations and rice aperitif and white spirit typically in the Chinese population [4]. In addition, although regular drinking is prevalent, binge drinking is the predominant pattern in China, especially in liquor drinkers. Indeed, according to a recent national survey, 29.8% of male drinkers drank heavily once a week or more and 83.6% of them drank heavily occasionally [5].

We believe that liquor drinking, especially the binge drinking pattern, is particularly deleterious to health. One of the difficulties in drinking studies is that drinkers may stop drinking when they become unhealthy or have a severe disease diagnosed. Under such circumstances, the risk of drinking would be underestimated. We hypothesize that in the elderly past drinkers and liquor drinkers particularly have increased risks of cardiovascular and non-cardiovascular diseases. Such research requires a study population with a high proportion of drinkers including both past and current drinkers. We recently conducted an elderly population-based study in Shanghai, China. The proportion of alcohol intake was high in men [6–9]. In the present study, we investigated the association between the quantity and type of alcoholic beverage consumption and the risk of cardiovascular and non-cardiovascular mortality in this elderly male Chinese population.

## Methods

### Study population

Our study was conducted in the framework of the Chronic Disease Detection and Management in the Elderly (≥60 years of age) Program supported by the municipal government of Shanghai, as described previously [6–9]. In a newly urbanized suburban town 30 km from the city center, we invited all residents of at least 60 years of age to participate in comprehensive examinations of cardiovascular disease and risk. All study participants were previously doing farming in their villages, and retired when they participated in the study. The Ethics Committee of Ruijin Hospital, Shanghai Jiao Tong University School of Medicine, approved the study protocol. All subjects provided written informed consent.

A total of 3997 subjects (participation rate, 90%) were enrolled in the period from 2006 to 2011 and followed up for vital status and cause of death till June 30, 2013. We excluded 2212 women because of low drinking rate (1.3%, *n* = 29), and further excluded 85 men from the present analysis because information on alcohol intake was not recorded. Thus, the number of participants included in the present analysis was 1702.

### Field work

The methods for field work had been described previously [6–9]. One experienced physician measured each participant’s blood pressure three times consecutively on the nondominant arm using a validated Omron 7051 oscillometric blood pressure monitor (Omron Healthcare, Kyoto, Japan) after the subjects had rested for at least 5 min in the sitting position. These three blood pressure readings were averaged for analysis. Hypertension was defined as a sitting blood pressure of at least 140 mmHg systolic or 90 mmHg diastolic or as the use of antihypertensive drugs.

A trained technician performed anthropometric measurements, including body height and body weight. Body mass index was calculated as the body weight in kilograms divided by the body height in meters squared. Abdominal obesity was defined as a waist circumference ≥ 90 cm.

Venous blood samples were drawn after overnight fasting for the measurement of plasma glucose and serum total cholesterol and triglycerides. Diabetes mellitus was defined as a plasma glucose level of at least 7.0 mmol/L while fasting or 11.1 mmol/L at any time or as the use of antidiabetic agents.

### Alcohol consumption

A standardized questionnaire was administered by a physician to collect information on medical history, alcohol consumption, smoking habits and the use of medications. Alcohol consumption was assessed for the quantity and frequency of various alcoholic beverages consumed during the past 12 months. Participants were inquired about the alcohol use, type of alcoholic beverages (beer, wine, aperitif or liquor), and frequency and quantity of consumption per week*.* Beers (4–6% v/v ethanol) and wines (10–12% v/v ethanol) are usually in bottles of 750 ml. Yellow or white rice aperitifs (15–18% v/v ethanol) and hard liquors (52–53% v/v ethanol) are in bottles of 500 ml. We estimated that one bottle of beer, wine, aperitif, and liquor on average contained 30, 90, 90, and 200 g of ethanol, respectively [10]. Average alcohol consumption (in grams per week) was computed by multiplying frequency and amount of the alcoholic beverages consumed. Individuals were classified as non-drinkers, past drinkers (stopped drinking for at least 12 months), and current light-to-moderate (1 to 299 g/week) and heavy drinkers (≥300 g/week) [10, 11]. Because beer and wine are considered similarly as low volume alcohol in China, we classified beer and wine drinkers as a single group in the analysis according to the type of beverages.

### Follow-up

Information on vital status and the cause of death was obtained from the official death certificate, with further confirmation by the local Community Health Center and family members of the deceased people, as described previously [6–9]. The International Classification of Diseases Ninth Revision (ICD-9) was used to classify the cause of death. Cardiovascular mortality included deaths attributable to stroke, myocardial infarction, and other cardiovascular diseases (ICD-9, 390.0–459.9).

### Statistical methods

For database management and statistical analysis, we used SAS software (version 9.4; SAS Institute, Cary, NC, USA). Means were compared by the analysis of variance (ANOVA) with the Student-Newman-Keuls test for a posteriori between-group contrast at a significance level set at 5%. Proportions were compared by the Chi-square test. Continuous measurements with a skewed distribution were expressed as median with interquartile range and were analyzed using the non-parametric Kruskal-Wallis test. The log-rank test was used to compare the cumulative incidence of mortality between groups with the Kaplan-Meier survival function to show the time to death. Multiple Cox regression analysis was performed to compute hazard ratios (HRs) and 95% confidence intervals (CIs) for the association between alcohol intake and mortality. Proportional hazards assumption was checked by assessing the Schoenfeld residuals [12]. A two-tailed *P* value less than 0.05 was considered statistically significant.

## Results

### Characteristics of the study participants

The 1702 male study participants included 843 (49.5%) non-drinkers, 241 (14.2%) past drinkers, and 618 (36.3%) current light-to-moderate (*n* = 241, 14.2%) and heavy drinkers (*n* = 377, 22.1%). In current drinkers, the number of beer/wine, aperitif and liquor/mix drinkers was 203 (32.8%), 273 (44.2%), and 142 (23.0%), respectively.

The study participants differed significantly (*P* ≤ 0.02) across the alcohol consumption categories in most of the baseline characteristics, such as age (67.4 to 69.3 years), systolic/diastolic blood pressure (135.7 to 142.9/80.2 to 83.9 mmHg), current smoking (45.2 to 67.9%), prevalence of hypertension (56.9 to 66.0%), and the use of antihypertensive drugs (32.6 to 54.4%). Nonetheless, they had similar (*P* ≥ 0.12) body-mass index (23.5 kg/m^2^), waist circumference (81.1 cm), prevalence of central obesity (19.6%), pulse rate (74.6 beats/min), serum total cholesterol (5.45 mmol/L) and triglycerides (1.55 mmol/L), and the prevalence of diabetes mellitus (7.6%, Table 1).

**Table 1:** Characteristics of the study participants by alcohol intake (*n* = 1702)

Characteristics	Non-drinkers (***n*** = 843)	Past drinkers (***n*** = 241)	Current drinkers (***n*** = 618)	
			Light-to-moderate (n = 241)	Heavy (n = 377)
Age, years	69.2 ± 7.4	69.3 ± 7.0	67.8 ± 7.0*	67.4 ± 6.6*
Body mass index, kg/m^2^	23.4 ± 3.5	23.5 ± 3.6	23.4 ± 3.1	23.8 ± 3.5
Waist circumference, cm	80.5 ± 9.4	81.2 ± 9.8	81.4 ± 9.7	81.9 ± 10.0
Central obesity, n (%)	150 (17.8)	48 (19.9)	45 (18.7)	88 (23.3)*
Systolic blood pressure, mmHg	136.7 ± 19.6	135.7 ± 19.6	138.7 ± 19.4	142.9 ± 19.8*
Diastolic blood pressure, mmHg	80.5 ± 10.8	80.2 ± 10.5	82.2 ± 11.2	83.9 ± 11.0*
Pulse rate, beats/min	74.9 ± 11.8	74.6 ± 12.4	74.3 ± 12.1	74.0 ± 11.8
Fasting plasma glucose, mmol/l	5.24 ± 1.09	5.31 ± 1.09	5.19 ± 0.74	5.36 ± 1.15
Serum total cholesterol, mmol/l	5.38 ± 1.42	5.52 ± 1.42	5.47 ± 1.35	5.55 ± 1.46
Serum triglycerides, mmol/l	1.55 (1.17–1.70)	1.56 (1.16–1.72)	1.55 (1.10–1.72)	1.58 (1.14–1.83)
Current smoking, n (%)	413 (49.0)	109 (45.2)	146 (60.6)*	256 (67.9)*
Hypertension, n (%)	480 (56.9)	159 (66.0)*	154 (63.9)*	239 (63.4)*
Use of antihypertensive drugs, n (%)	318 (37.7)	131 (54.4)*	90 (37.3)	123 (32.6)*
Diabetes mellitus, n (%)	67 (8.0)	22 (9.1)	12 (5.0)	29 (7.7)

Values are mean ± standard deviations, median (interquartile range) or number of participants (% of column total)

*P ≤ 0.05 vs. non-drinkers

### Alcohol intake and mortality

During a median follow-up of 5.9 years (interquartile range, 4.9–6.8 years), the cumulated number of person-years was 8955, and all-cause, cardiovascular and non-cardiovascular deaths occurred in 211, 98 and 113 subjects, respectively. The corresponding incidence rates were 23.6/1000, 10.9/1000 and 12.6/1000 person-years, respectively.

**Fig. 1:**
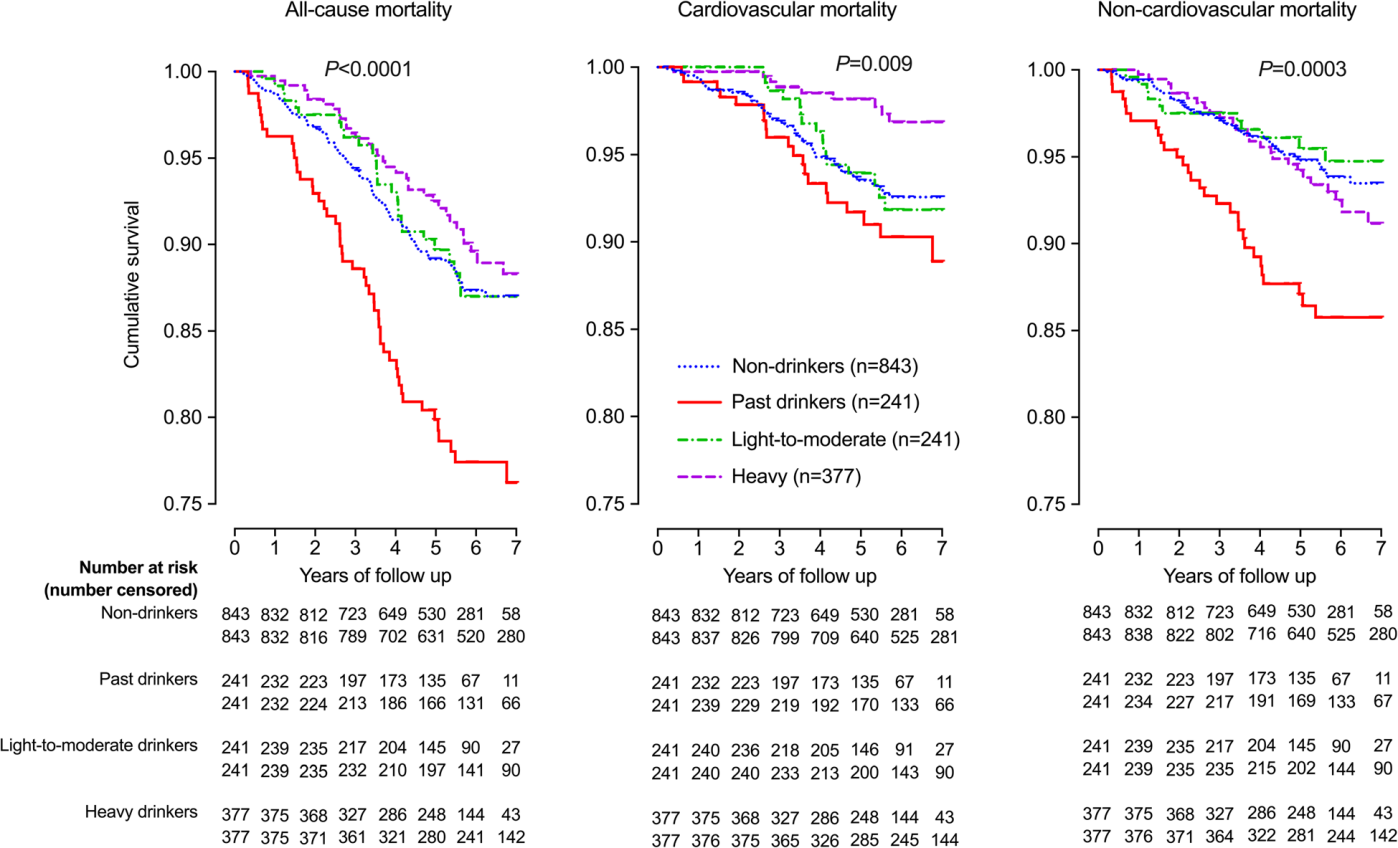
Kaplan-Meier survival curve for all-cause, cardiovascular and non-cardiovascular mortality according to alcohol intake. The number of participants is given for each category of alcohol intake. The *P* value for log-rank test is also given

Kaplan-Meier survival analyses showed a significant difference in the incidence of total, cardiovascular and non-cardiovascular mortality between non-drinkers, past drinkers and current light-to-moderate and heavy drinkers, with the highest incidence in past drinkers (log-rank test, *P* ≤ 0.009, Fig. 1). After adjustment for age, body-mass index, current smoking, serum total cholesterol and triglycerides, fasting plasma glucose, and the prevalence of hypertension and diabetes mellitus, compared with non-drinkers, past drinkers, but not light-to-moderate or heavy drinkers (*P* ≥ 0.32), had a significantly higher risk of total (HR 1.90, 95% CI 1.35–2.68, *P* = 0.0003) and non-cardiovascular mortality (HR 2.46, 95% CI 1.55–3.91, *P* = 0.0001, Table 2). The risk of cardiovascular mortality was highest in past drinkers and lowest in heavy drinkers. Statistical significance relative to non-drinkers, however, was not achieved in either group (*P* ≥ 0.07).

**Table 2:** All-cause, cardiovascular and non-cardiovascular mortality in relation to alcohol intake

	Non-drinkers	Past drinkers	Current drinkers	
			Light-to-moderate	Heavy
Number of participants	843	241	241	377
Number of person-years	4429	1201	1311	2014
All-cause mortality				
Number of deaths	100	50	27	34
Rate per 1000 person-years	22.6	41.6	20.6	16.9
Age-adjusted HR (95% CI)	1	1.89 (1.35–2.66)	1.07 (0.70–1.63)	0.92 (0.62–1.36)
Multivariate-adjusted HR (95% CI)	1	1.90 (1.35–2.68)	1.09 (0.71–1.67)	0.89 (0.60–1.34)
Cardiovascular mortality				
Number of deaths	53	20	16	9
Rate per 1000 person-years	12.0	16.7	12.2	4.5
Age-adjusted HR (95% CI)	1	1.50 (0.90–2.51)	1.25 (0.72–2.19)	0.51 (0.25–1.02)
Multivariate-adjusted HR (95% CI)	1	1.44 (0.85–2.44)	1.17 (0.66–2.07)	0.51 (0.25–1.05)
Non-cardiovascular mortality				
Number of deaths	47	30	11	25
Rate per 1000 person-years	10.6	25.0	8.4	12.4
Age-adjusted HR (95% CI)	1	2.36 (1.50–3.74)	0.88 (0.45–1.69)	1.33 (0.81–2.16)
Multivariate-adjusted HR (95% CI)	1	2.46 (1.55–3.91)	0.92 (0.48–1.79)	1.29 (0.78–2.13)

The multivariate analyses were adjusted for age, body mass index, current smoking, serum total cholesterol and triglycerides, fasting plasma glucose and the prevalence of hypertension and diabetes mellitus at baseline

CI: confidence interval; HR: hazard ratio

### Alcoholic beverages and mortality

**Fig. 2:**
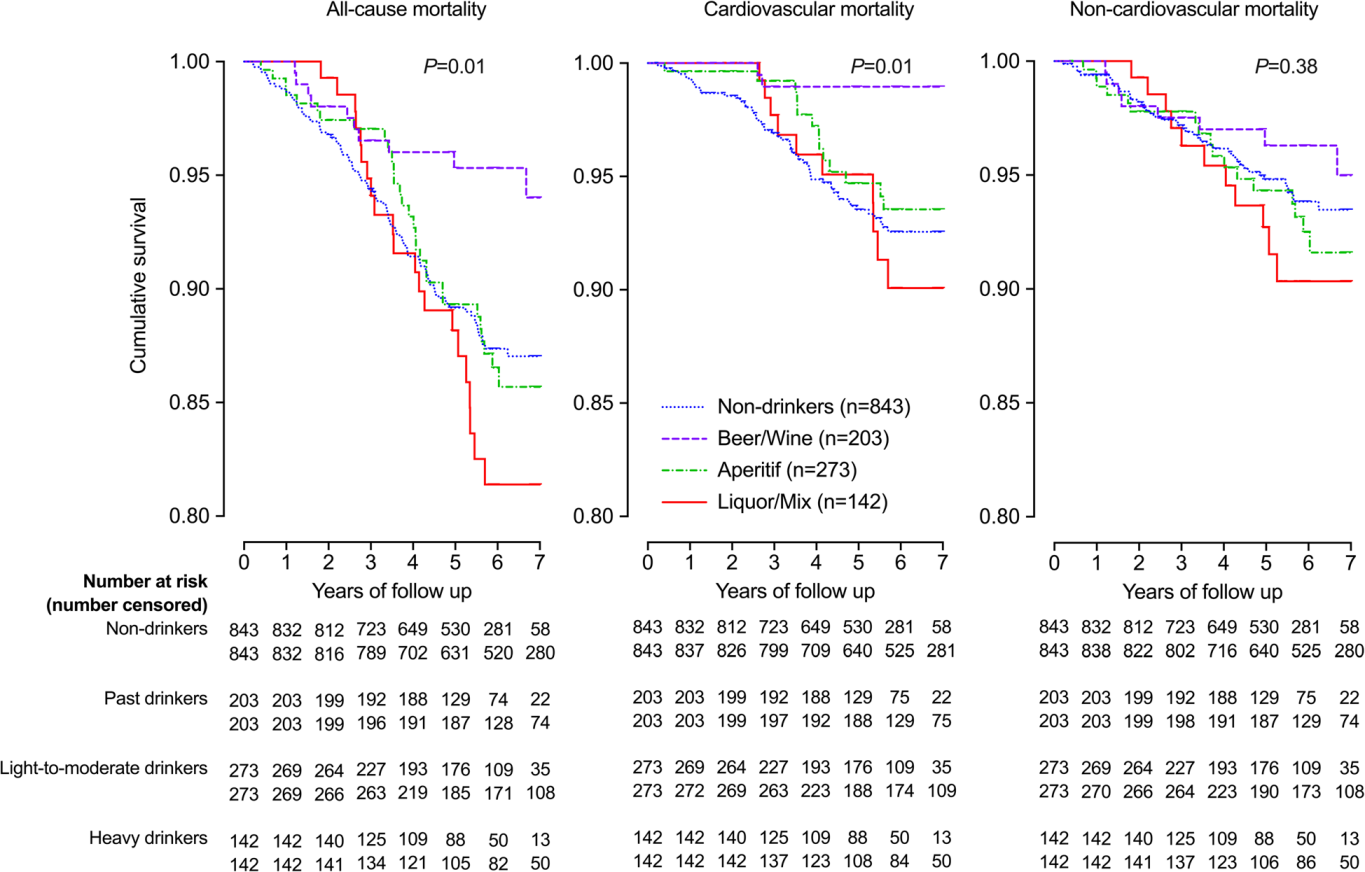
Kaplan-Meier survival curve for all-cause, cardiovascular disease and non-cardiovascular disease mortality according to alcoholic beverages. The number of participants is given for non-drinkers and drinkers of various alcoholic beverages. The *P* value for log-rank test is also given

Further analyses in current drinkers showed significant differences in the incidence of total and cardiovascular mortality between non-drinkers and current beer/wine, aperitif, and liquor/mix drinkers, with the highest incidence in liquor/mix drinkers and lowest incidence in beer/wine drinkers (log-rank test, *P* = 0.01, Fig. 2). Similar trends were observed for non-cardiovascular mortality, though statistical significance was not achieved (*P* = 0.38). After adjustment for total alcohol consumption and above-mentioned confounding factors, liquor/mix drinkers, but not beer/wine or aperitif drinkers, had a significantly higher risk of cardiovascular mortality (HR 3.23, 95% CI 1.24–8.39, *P* = 0.02) than non-drinkers (Table 3).

**Table 3:** All-cause, cardiovascular and non-cardiovascular mortality in relation to the type of alcoholic beverages

	Non-drinkers	Current drinkers		
		Beer/wine	Aperitif	Liquor/mix
Number of participants	843	147/56	273	142
Number of person-years	4429	834/319	1424	748
Ethanol intake (g/week)	–	210 (182–420)	504 (252–630)	600 (280–840)
All-cause mortality				
Number of deaths	100	10	30	21
Rate per 1000 person-years	22.6	8.7	21.1	28.1
Age-adjusted HR (95% CI)	1	0.53 (0.28–1.02)	1.04 (0.69–1.57)	1.52 (0.95–2.44)
Multivariate-adjusted HR (95% CI)	1	0.53 (0.27–1.06)	1.00 (0.60–1.67)	1.50 (0.82–2.72)
Cardiovascular mortality				
Number of deaths	53	2	13	10
Rate per 1000 person-years	12.0	1.7	9.1	13.4
Age-adjusted HR (95% CI)	1	0.23 (0.06–0.94)	0.87 (0.47–1.59)	1.46 (0.74–2.88)
Multivariate-adjusted HR (95% CI)	1	0.36 (0.08–1.56)	1.50 (0.69–3.30)	3.23 (1.24–8.39)
Non-cardiovascular mortality				
Number of deaths	47	8	17	11
Rate per 1000 person-years	10.6	6.9	11.9	14.7
Age-adjusted HR (95% CI)	1	0.80 (0.38–1.70)	1.22 (0.70–2.12)	1.59 (0.82–3.06)
Multivariate-adjusted HR (95% CI)	1	0.71 (0.32–1.56)	0.90 (0.46–1.77)	1.23 (0.58–2.64)

The multivariate analyses were adjusted for age, body-mass index, current smoking, serum total cholesterol and triglycerides, fasting plasma glucose, total alcohol consumption (ethanol intake per week) and the prevalence of hypertension and diabetes mellitus at baseline

CI: confidence interval; HR: hazard ratio

With similar adjustments applied as above, compared with the current beer/wine drinkers, liquor/mix drinkers had a significantly higher risk of all-cause (HR 3.07, 95% CI 1.39–6.79, *P* = 0.006) and cardiovascular mortality (HR 10.49, 95% CI 2.00–55.22, *P* = 0.006). Similar trends were observed for non-cardiovascular mortality (HR 1.94, 95% CI 0.73–5.16, *P* = 0.18).

## Discussion

The key finding of our study was that past drinkers had a higher risk of all-cause and non-cardiovascular mortality. With regard to the alcoholic beverages, liquor/mix drinkers tended to have a higher risk of mortality, especially when compared with beer/wine drinkers.

Our observation that past drinkers had a higher risk of all-cause mortality is in keeping with the results of several previous studies [13, 14]. In an American prospective study, past drinkers (*n* = 24,904) had a higher risk of all-cause (HR 1.35, 95% CI 1.02–1.11) and cardiovascular (HR 1.27, 95% CI 1.17–1.37) mortality than lifetime abstainers (*n* = 76,869) [13]. In a Spanish study, past drinkers (*n* = 432) also had a higher risk of all-cause mortality than never drinkers (*n* = 941) both before (HR 1.49, 95%CI 1.03–2.14) and after exclusion of participants with functional limitations (*n* = 2665, HR 1.68, 95% CI 1.08–2.60) [14]. ﻿In the Prospective Urban Rural Epidemiology (PURE) study, however, past drinkers (*n* = 4255), compared with never drinkers (*n* = 74,685), had a significantly higher risk of all-cause mortality (HR 1.56, 95%CI 1.27–1.92) but non-significantly higher risk of fatal and nonfatal cardiovascular disease (HR 1.19, 95%CI 0.94–1.50) [15]. In addition, the risk in 1207 past drinker versus 1076 never drinkers was not significantly higher either for all-cause (HR 1.13, 95%CI 0.91–1.41) or cardiovascular mortality (HR 1.08, 95%CI 0.77–1.53) in a group of patients with a previous myocardial infarction, angina, or stroke enrolled in the UK Biobank Study [16]. The risk of past drinking might have been confounded by the existence of severe diseases.

One of the possible mechanisms for the higher disease and mortality risk of past drinkers is that past drinkers often stopped alcohol intake because of illnesses or health concerns [17]. Indeed, in our present study, past drinkers, compared with non-drinkers and current drinkers, had a significantly higher prevalence of hypertension and use of antihypertensive drugs and a slightly higher prevalence of diabetes mellitus.

In line with the results of several recent studies [14, 18, 19], our study did not show any beneficial effect of alcohol intake on cardiovascular disease mortality. In the abovementioned Spanish prospective study, neither light (1.43–20 g/d for men and < 10 g/d for women, *n* = 785) nor moderate (20–40 g/d for men and 10–24 g/d for women, *n* = 505) drinkers had a lower risk of mortality than never drinkers [14]. However, these recent observations are in contradiction to the cardiovascular protective effects observed in several earlier previous studies [13, 20–22]. The latter observation might have been confounded by classifying past drinkers as abstainers. In a recent meta-analysis of 87 cohort studies (*n* = 3,998,626), after controlling for abstainer bias, light drinking (1.3–24.9 g/day) did not show any protective effect on the risk of mortality (relative risk 0.97, 95% CI 0.88–1.07) [18]. Similarly, in a cohort study in 24,029 US adults aged more than 50 years, low level of moderate drinking (< 7 drinks/week) had a similar risk of all-cause mortality as occasional drinkers (HR 1.02, 95% CI 0.94–1.11), whereas high level of moderate drinking (7 to < 14 drinks/week) had a significantly higher risk of all-cause mortality than occasional drinkers (HR 1.14, 95% CI 1.02–1.28) [19].

Abstaining might also have confounded the results of our study on the risks of heavy drinking. Heavy drinkers more likely stop drinking, because of illnesses or health concerns. In fact, current heavy drinkers had a higher systolic and diastolic blood pressure than non-drinkers, past drinkers and current light-to-moderate drinkers. The harmful effect of high blood pressure may appear with the longer-term follow-up.

Our observation on the lower risk of cardiovascular disease mortality in the current wine/beer drinkers is in accordance with the results of numerous previous studies [23–25]. Indeed, in a recent cohort study (*n* = 114,970), wine (HR 0.88, 95% CI 0.69–1.13) and beer drinkers (HR 0.86, 95% CI 0.67–1.09), but not spirit or liquor drinkers (HR 0.97, 95% CI 0.81–1.16), tended to have lower risks of all-cause mortality [15]. Our study only included a small number of wine drinkers (*n* = 56). The number of events in wine drinkers was small (n = 1) and does not allow analysis in beer and wine drinkers separately.

Our study should be interpreted within the context of its limitations. First, we only collected information on the amount and type of alcoholic beverages at baseline but not during follow-up. Second, the quantity of alcohol consumption was self-reported. Over- or under-reporting is possible. Third, we did not collect information on the type of alcohol beverages or the lifetime drinking in past drinkers. We therefore were unable to perform analysis according to various alcoholic beverages and cumulative exposure to the risk in the past drinkers. Fourth, for the comparison between various alcoholic beverages, the number of events was small, leading to wide confidence intervals of the hazard ratios. Finally, because of the retrospective nature of the present analysis, sample size estimation was not performed prior to the analysis. Nonetheless, according to the observed proportion of alcohol intake, the present study may provide 84.9% of power to detect the observed association between alcohol intake and total mortality.

## Conclusions

In summary, our study showed risks of mortality associated with past drinking and liquor drinking in men aged over 60 years of age in Shanghai, China. In fact, these two drinking factors are closely related. It is possible that high ethanol concentration of liquor drinking confers risks and when there are health concerns motivates abstaining from alcohol drinking. The latter hypothesis should be investigated in future studies of larger sample size and with information on the type of alcoholic beverages in past drinkers.

## Abbreviations

CI: confidence interval
HR: hazard ratio
